# Supplementary material for: The impact of phenotypic heterogeneity of tumour cells on treatment and relapse dynamics
Source: PLoS Comput Biol. 2021 Feb 12;17(2):e1008702. doi: 10.1371/journal.pcbi.1008702 (PMC7906468; doi:10.1371/journal.pcbi.1008702)
Supplement: S1 Text — (PDF) [file pcbi.1008702.s001.pdf]

S1 Text - Derivation of the stochastic model

Here we consider a stochastic model of tumour growth with two phenotypes, namely, slow and fast proliferating tumour cells. These subpopulations grow and switch their phenotypes at different rates. When the trait-dependent treatment is applied, they die at rates proportional to their growth rate. The trait-independent treatment induces the same mortality rate on both phenotypes. We start the derivation of the stochastic model by presenting these processes as a set of reactions that individual cells perform. The reactions are accompanied by corresponding reaction rates ρ_k and the change vector $\boldsymbol{\mu}_k$, that describes the effect of a single reaction. The reactions can be implemented in a stochastic simulation algorithm [1].

Reaction	Rate	Change vector
$S \longrightarrow S + S$	$\rho_1 = r_S \; n_S$	$\boldsymbol{\mu}_1 = (1, 0)^T$
$F \longrightarrow F + F$	$\rho_2 = r_F \; n_F$	$\boldsymbol{\mu}_2 = (0, 1)^T$
$S \longrightarrow F$	$\rho_3 = r_S \; p_F \; n_S$	$\boldsymbol{\mu}_3 = (-1, 1)^T$
$F \longrightarrow S$	$\rho_4 = r_F \; p_S \; n_F$	$\boldsymbol{\mu}_4 = (1, -1)^T$
$S \longrightarrow \phi$	$\rho_5 = \delta \; r_S \; n_S$	$\boldsymbol{\mu}_5 = (-1, 0)^T$
$F \longrightarrow \phi$	$\rho_6 = \delta \; r_F \; n_F$	$\boldsymbol{\mu}_6 = (0, -1)^T$
$S \longrightarrow \phi$	$\rho_7 = m \; n_S$	$\boldsymbol{\mu}_7 = (-1, 0)^T$
$F \longrightarrow \phi$	$\rho_8 = m \; n_F$	$\boldsymbol{\mu}_8 = (0, -1)^T$

Table A The eight possible reactions in the stochastic model. Reactions going to ϕ represent cell death events. S and F represent individual cells, n_S and n_F are their total numbers in the cancer cell population. Reaction k occurs with rate ρ_k and changes the cell number vector $\boldsymbol{n} = (n_S, n_F)$ by the change vector $\boldsymbol{\mu}_k$.

As this infinitesimal time element tends to zero the discrete model leads to a stochastic differential equation model [2, Section 5.1]. To get there, we compute the vector of expected change $\boldsymbol{\mu}$ to the population vector $\boldsymbol{n}$ per time unit as the sum over all possible change vectors weighted by their respective rates,

$$\begin{aligned}\mathbb{E}\left[\boldsymbol{\mu}\right] &= \sum_{k=1}^{8} \rho_k \; \boldsymbol{\mu}_k \, \Delta t \\ &= \begin{bmatrix} r_S \; (1 - p_F - \delta) \; n_S + r_F \; p_S \; n_F - m \; n_S \\ r_F \; (1 - p_S - \delta) \; n_F + r_S \; p_F \; n_S - m \; n_F \end{bmatrix} \Delta t\end{aligned}$$

Similarly, we can obtain the covariance matrix of change rate to the population vector

$$\begin{aligned}\mathbb{E}\left[\boldsymbol{\mu}\,(\boldsymbol{\mu})^T\right] &= \sum_{k=1}^{8} \rho_k \; \boldsymbol{\mu}_k (\boldsymbol{\mu}_k)^T \, \Delta t \\ &= \begin{bmatrix} r_S \; (1 + p_F + \delta) \; n_S + r_F \; p_S \; n_F + m \; n_S & -r_S \; p_F \; n_S - r_F \; p_S \; n_F \\ -r_S \; p_F \; n_S - r_F \; p_S \; n_F & r_F \; (1 + p_S + \delta) \; n_F + r_S \; p_F \; n_S + m \; n_F \end{bmatrix} \Delta t\end{aligned}$$

If we now introduce a system size parameter V and convert the numbers of cells $\boldsymbol{n}$ to densities $\boldsymbol{x} = \frac{\boldsymbol{n}}{\mathrm{V}}$, we can write the expectation vector $\boldsymbol{a}(\boldsymbol{x})$, and the covariance matrix $\boldsymbol{B}(\boldsymbol{x})$ for the cell densities [2, Section 5.1],

$$\begin{aligned}\boldsymbol{a}(\boldsymbol{x}) &= \frac{1}{\Delta t}\,\mathbb{E}\left[\frac{\boldsymbol{\mu}}{\mathrm{V}}\right] = \begin{bmatrix} r_S \; (1 - p_F - \delta) \; x_S + r_F \; p_S \; x_F - m \; x_S \\ r_F \; (1 - p_S - \delta) \; x_F + r_S \; p_F \; x_S - m \; x_F \end{bmatrix}, \\ \boldsymbol{B}(\boldsymbol{x}) &= \frac{1}{\Delta t}\,\mathbb{E}\left[\frac{1}{\mathrm{V}^2} \; \boldsymbol{\mu}\,(\boldsymbol{\mu})^T\right] \\ &= \frac{1}{\mathrm{V}} \begin{bmatrix} r_S \; (1 + p_F + \delta) \; x_S + r_F \; p_S \; x_F + m \; x_S & -r_S \; p_F \; x_S - r_F \; p_S \; x_F \\ -r_S \; p_F \; x_S - r_F \; p_S \; x_F & r_F \; (1 + p_S + \delta) \; x_F + r_S \; p_F \; x_S + m \; x_F \end{bmatrix}\end{aligned}$$

We arrive at an approximate Fokker-Planck equation that can be written as

$$\partial_t P(\boldsymbol{x},t) = -\sum_{i=1}^{2} \partial_{x_i}[a_i(\boldsymbol{x})\ P(\boldsymbol{x},t)] + \frac{1}{2}\ \sum_{i=1}^{2}\sum_{j=1}^{2} \partial_{x_i}\partial_{x_j}[b_{ij}(\boldsymbol{x})\ P(\boldsymbol{x},t)]$$

with $a_i(\boldsymbol{x})$ and $b_{ij}(\boldsymbol{x})$ being the entries of $\boldsymbol{a}(\boldsymbol{x})$ and $\boldsymbol{B}(\boldsymbol{x})$. We see that the system size parameter V determines the relative contributions of the drift and diffusion terms in the Fokker-Planck equation, thus setting the relative effect of stochastic fluctuations on the system dynamics. Using the Feynmann-Kac Formula, we finally obtain a system of Itô stochastic differential equations from the Fokker-Planck equation,

$$d\boldsymbol{x} = \boldsymbol{a}(\boldsymbol{x})\ dt + \sqrt{\boldsymbol{B}(\boldsymbol{x})}d\boldsymbol{W} \tag{1}$$

where $\boldsymbol{W} = (W_1, W_2)^T$ consists of two independent Wiener processes W_i, or equivalently

$$d\boldsymbol{x} = \boldsymbol{a}(\boldsymbol{x})\ dt + \boldsymbol{C}(\boldsymbol{x})d\boldsymbol{W}$$

with $\boldsymbol{C}(\boldsymbol{x})^T\boldsymbol{C}(\boldsymbol{x}) = \boldsymbol{B}(\boldsymbol{x})$ [2, pp. 144]. This stochastic differential equation is numerically solved using the *sdeint* package (Matthew J. Aburn, version 0.2.1).

References

1. Sanft KR, Wu S, Roh M, Fu J, Lim RK, Petzold LR. StochKit2: Software for discrete stochastic simulation of biochemical systems with events. Bioinformatics. 2011;27(17):2457–2458.

2. Allen E. Modeling with Itô Stochastic Differential Equations. Springer; 2007.
