# Supplementary material for: The impact of phenotypic heterogeneity of tumour cells on treatment and relapse dynamics
Source: PLoS Comput Biol. 2021 Feb 12;17(2):e1008702. doi: 10.1371/journal.pcbi.1008702 (PMC7906468; doi:10.1371/journal.pcbi.1008702)
Supplement: S2 Text — (PDF) [file pcbi.1008702.s002.pdf]

S2 Text - Effect of the switching parameters

The switching parameters p_S and p_F determine the flux between adjacent subpopulations. Smaller values broaden the stable trait distribution and prolong the time until both treatment types have achieved the same tumour load reduction, as it takes longer for the slowest subpopulation to overtake the fastest subpopulation (Fig S2.1).

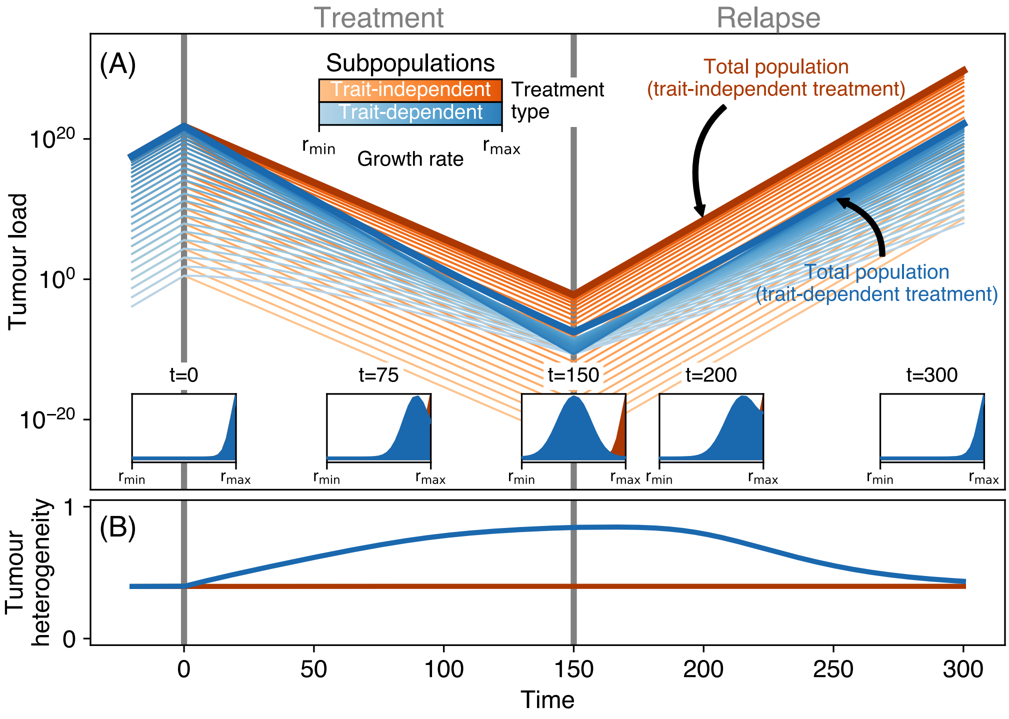

Figure S2.1 Same as Fig 2 but for slower switching between adjacent subpopulations, $p_S = p_F = 0.05$.

If the switching parameters are larger, the stable trait distribution is narrower, and both treatment types achieve equal tumour load reductions earlier (Fig S2.2). Throughout the paper, we have assumed that switching to adjacent subpopulations is equally likely. Asymmetric switching alters the stable trait distribution. If switching to faster-growing subpopulations is more likely than switching to slower-growing subpopulations, $p_F > p_S$, the trait distribution increases steeper towards faster growth rates (Fig S2.3). As subpopulations with larger growth rates are now more populated, also the growth rate-dependent treatment has a higher effect than for symmetric switching. Also, the realized growth rate of the fastest subpopulation is higher, which increases the tumour load and decreases the effect of the trait-independent treatment type. If switching to slower-growing subpopulations is more likely, the maximum of the trait distribution moves to slightly slower growing subpopulations, which decreases the realized growth rates and leads to lower tumour loads and therefore more effective treatment (Fig S2.4).

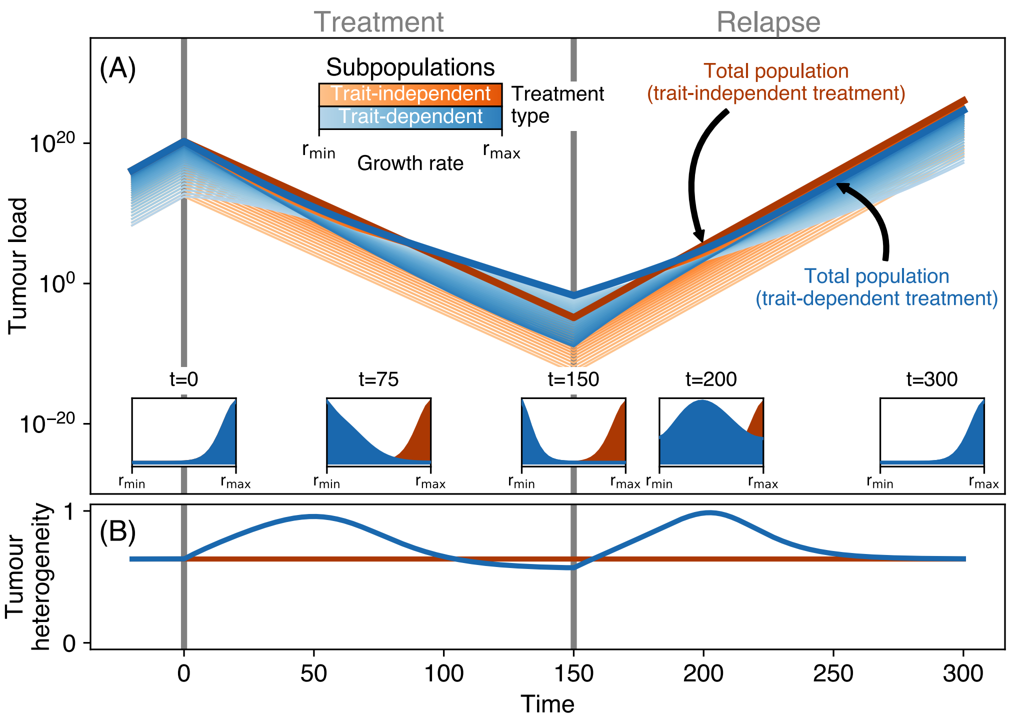

Figure S2.2 Same as Fig 2 but for faster switching between adjacent subpopulations, $p_S = p_F = 0.5$.

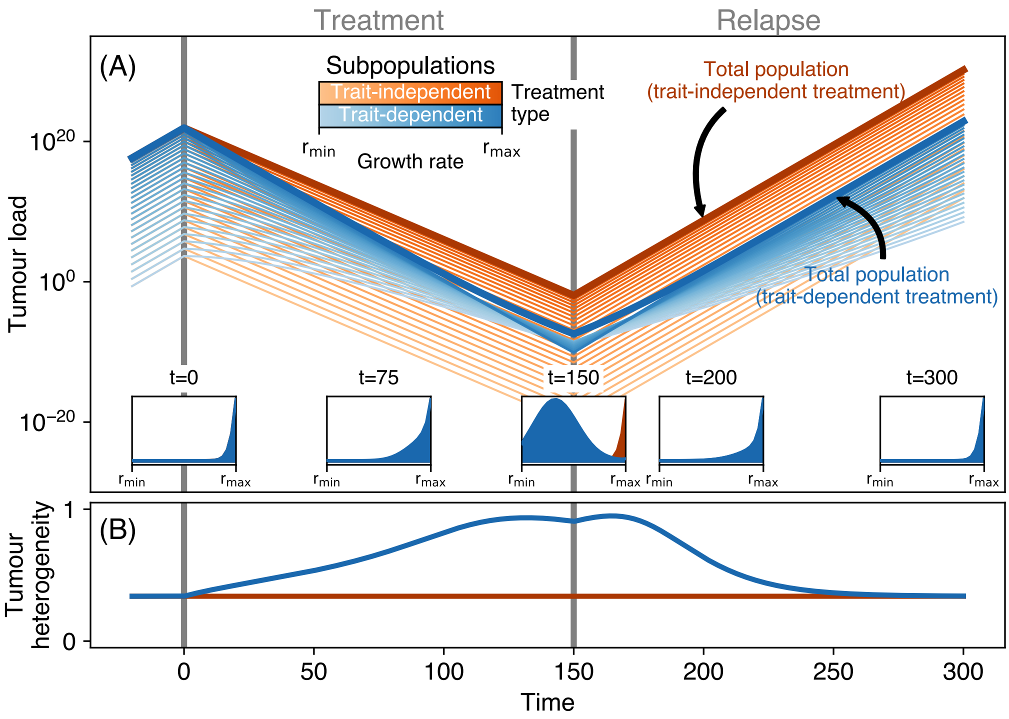

Figure S2.3 Same as Fig 2 but now for asymmetric switching between adjacent subpopulations, assuming an increased flux to faster-growing subpopulations, $p_S = 0.1$ and $p_F = 0.2$.

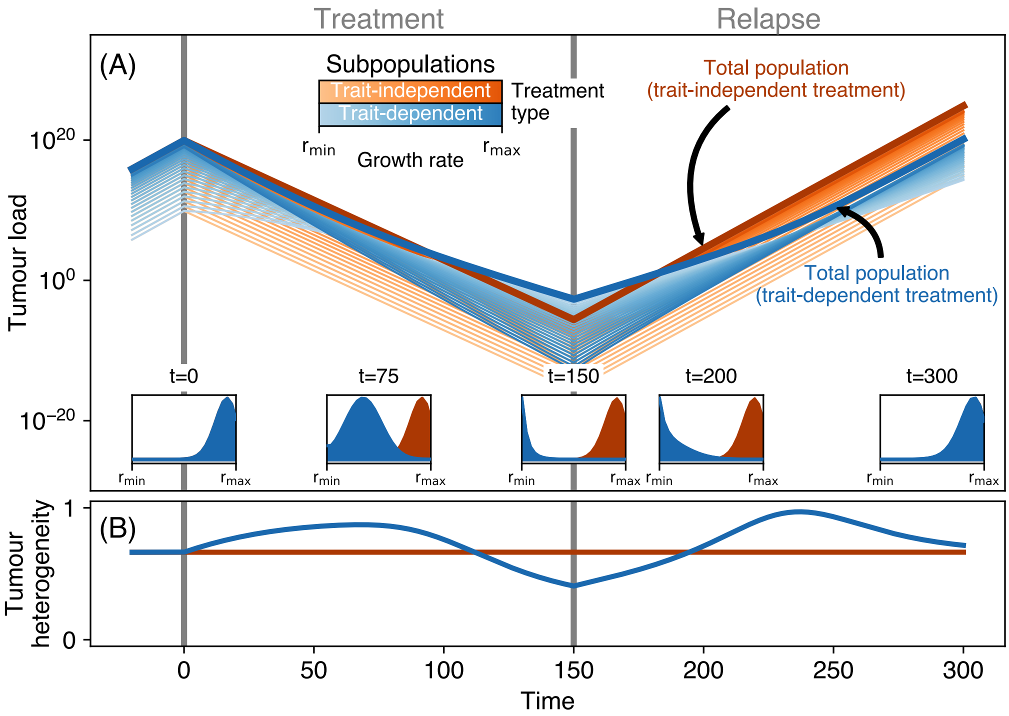

Figure S2.4 Same as Fig 2 but now for asymmetric switching between adjacent subpopulations, assuming a decreased flux to faster-growing subpopulations, $p_{\mathrm{S}} = 0.2$ and $p_{\mathrm{F}} = 0.1$.
