# Supplementary figures and images for: The impact of phenotypic heterogeneity of tumour cells on treatment and relapse dynamics
Source: PLoS Comput Biol. 2021 Feb 12;17(2):e1008702. doi: 10.1371/journal.pcbi.1008702 (PMC7906468; doi:10.1371/journal.pcbi.1008702)

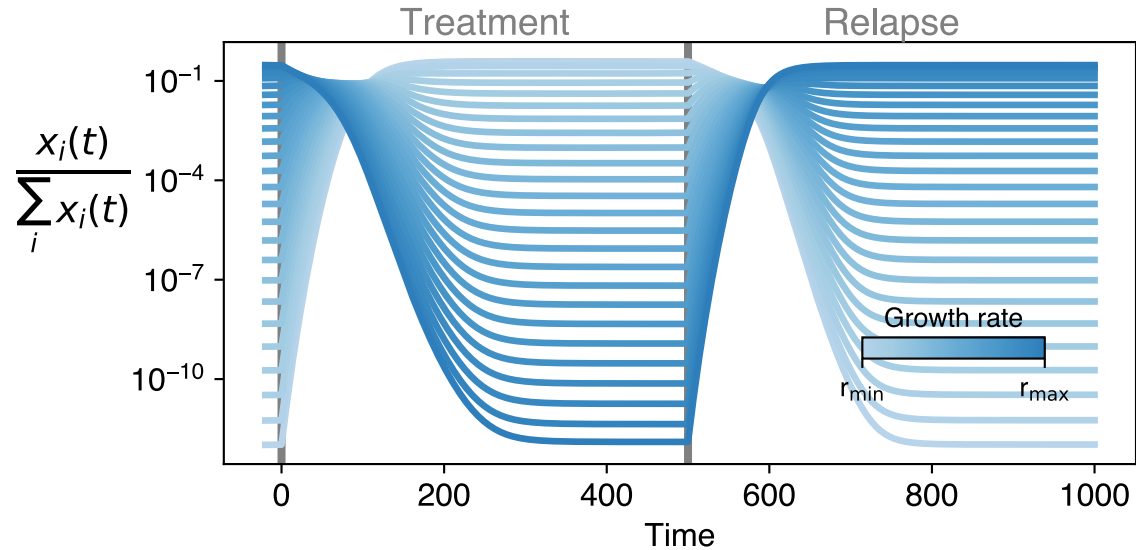

Supplement: S1 Fig — Our model gives rise to a stable trait distribution (see constant ratios prior to the treatment phase). The trait-dependent treatment type creates another stable trait distribution towards the end of the treatment phase, where the slowest-growing subpopulations dominate. Note that treatment phase and relapse phase are prolonged here compared to Fig 2 for better visualization. (PDF) [file pcbi.1008702.s003.pdf]

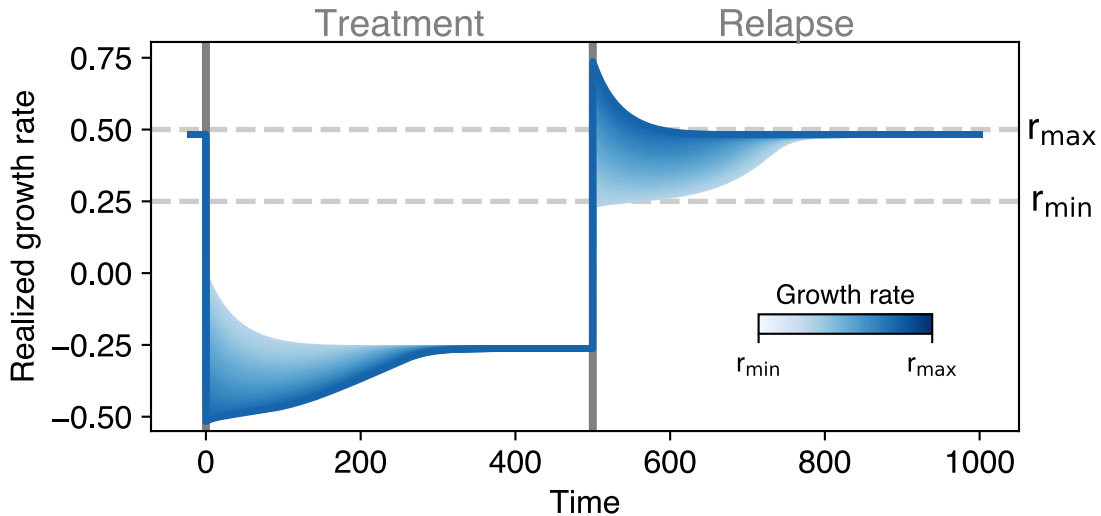

Supplement: S2 Fig — Switching to the adjacent slower subpopulation limits the realized growth rate of the fastest subpopulation to slightly below rmax. Note that treatment phase and relapse phase are prolonged here compared to Fig 2 for better visualization. (PDF) [file pcbi.1008702.s004.pdf]

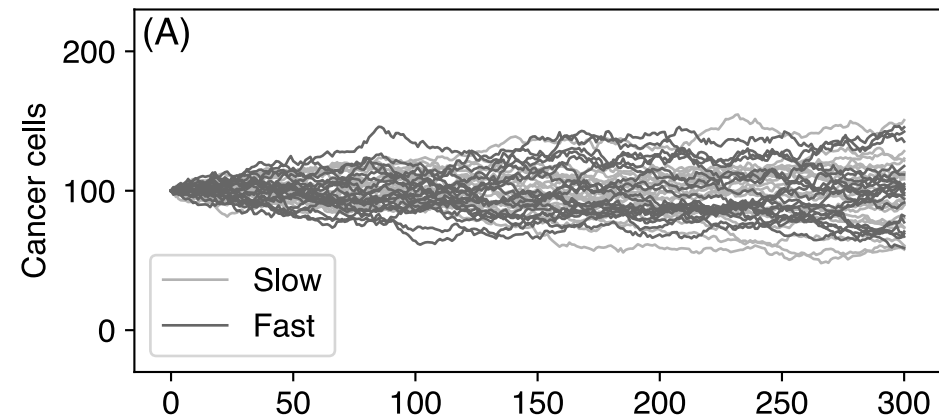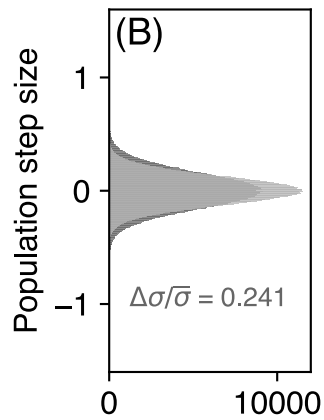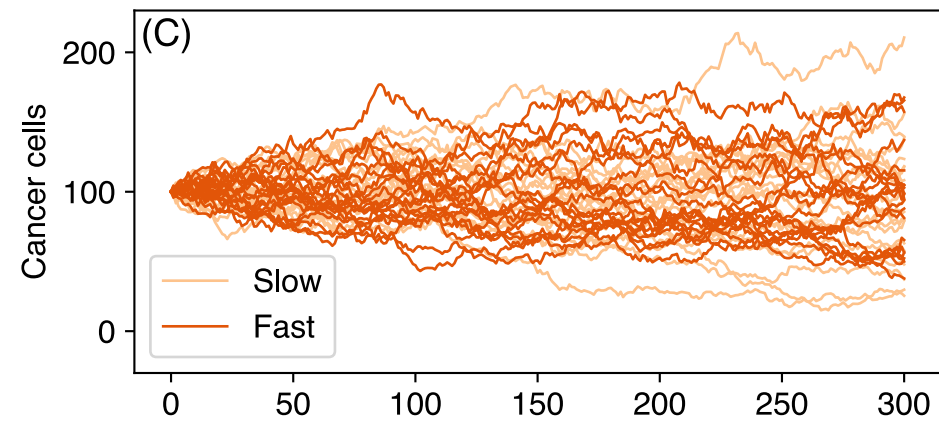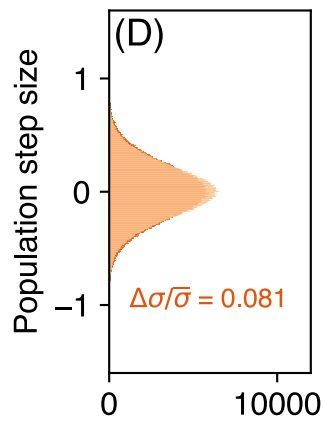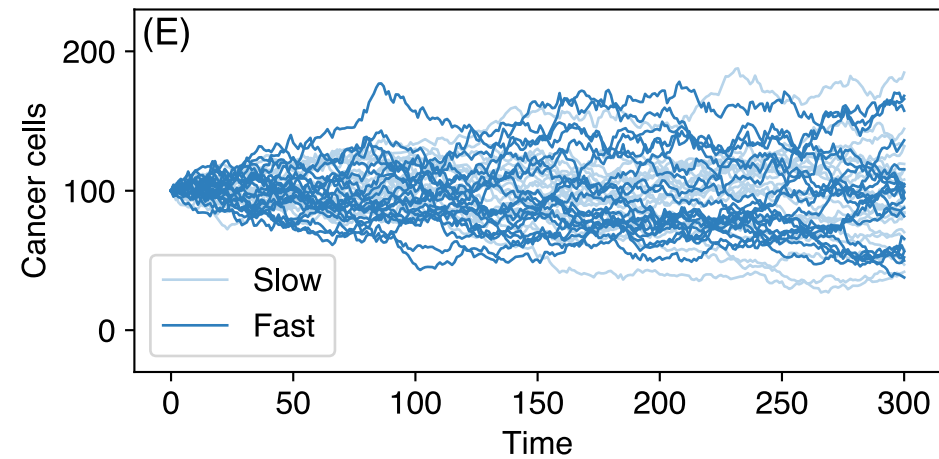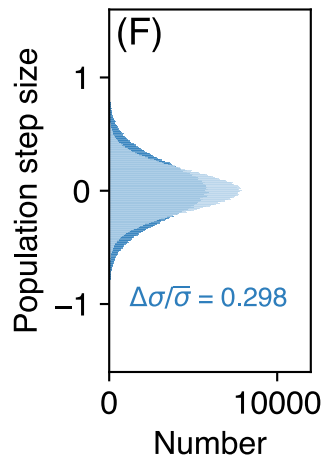

Supplement: S3 Fig — The left column shows the time series for 20 replicates. The right column visualizes the population step sizes taken in the simulation (numerical solver evaluation intervals dt = 0.01, plotting time interval 100dt). We use the normalized difference of the standard deviation of the slow and fast subpopulations Δσ/σ¯ to characterize the different widths of the step size distributions. Large values indicate that the changes of the slow subpopulation are on average smaller than the changes of the fast subpopulation. (PDF) [file pcbi.1008702.s005.pdf]

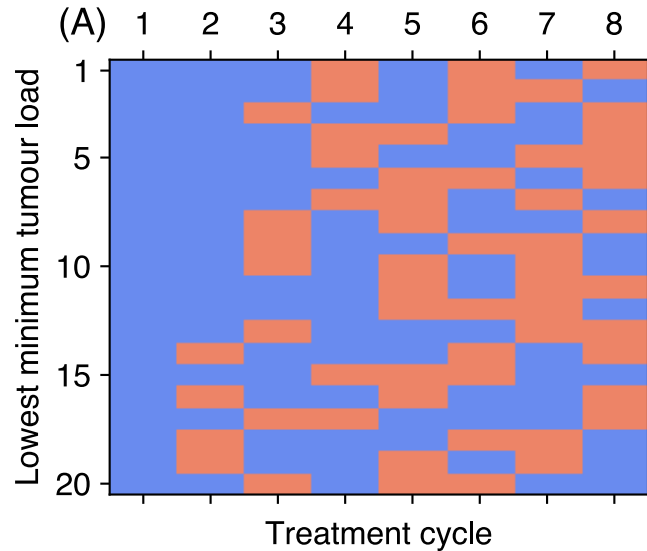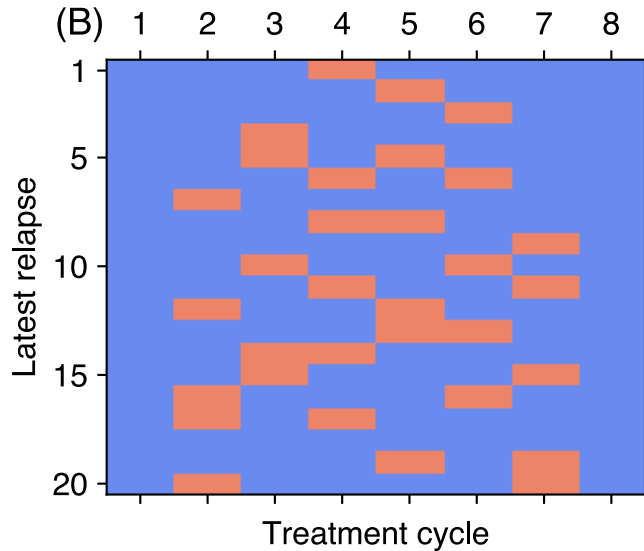

Supplement: S4 Fig — Best sequences are at the top, trait-dependent treatment type intervals are blue, trait-independent treatment type intervals are orange. We allowed for 8 different treatment intervals and investigated all 256 combinations. (PDF) [file pcbi.1008702.s006.pdf]

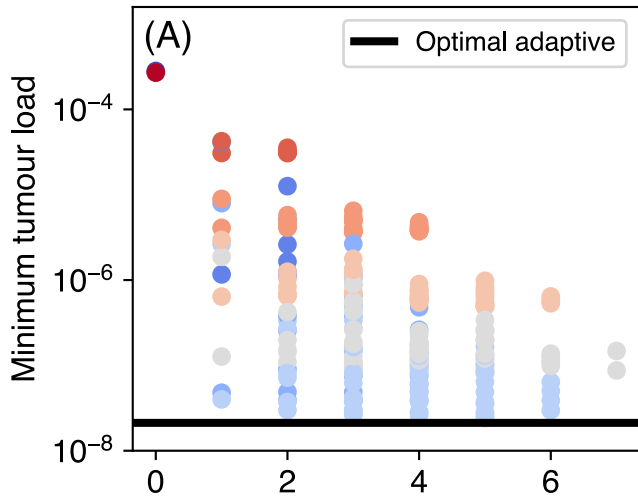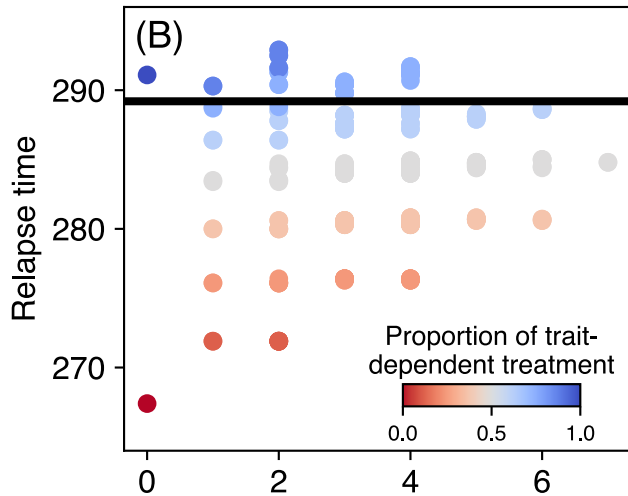

Number of treatment changes

Supplement: S5 Fig — A maximum of 7 treatment alterations are possible. The blue-to-red colour gradient indicates the proportion of trait-dependent treatment type in every treatment pattern. Note that Fig 5 shows the correlation of minimum tumour load and relapse time. (PDF) [file pcbi.1008702.s007.pdf]

Minimum tumour load

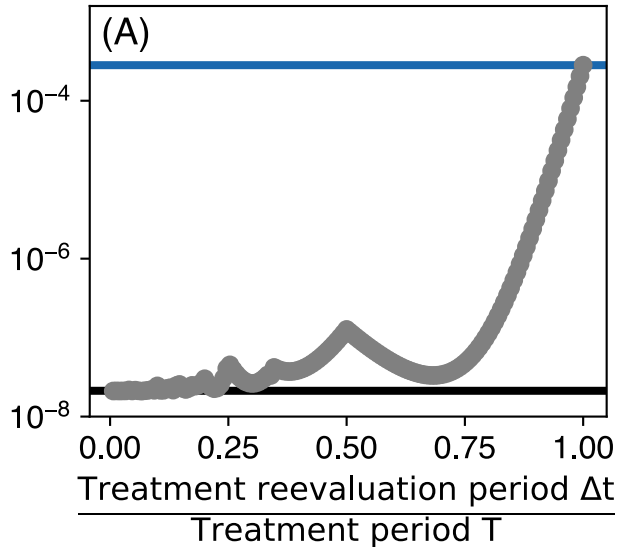

Relapse time

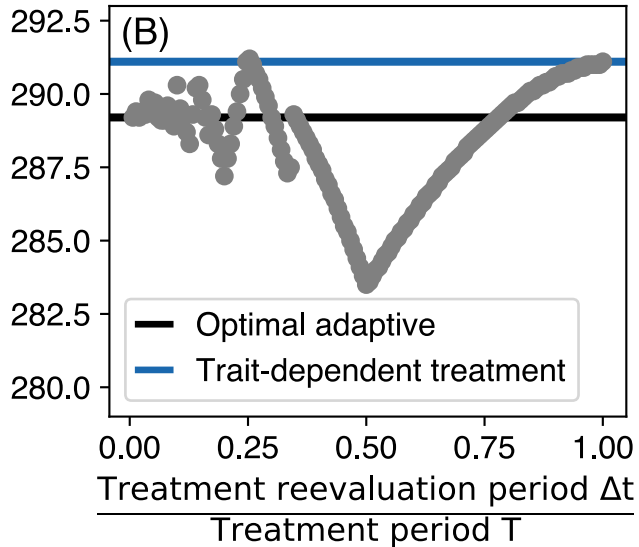

Supplement: S6 Fig — Δt/T → 0 corresponds to the optimal adaptive treatment, whereas Δt/T ≥ 1 results in only trait-dependent treatment. The discontinuities arise at reevaluation periods where the number of possible treatment alterations changes. Note that Fig 5 shows the correlation of minimum tumour load and relapse time. (PDF) [file pcbi.1008702.s008.pdf]

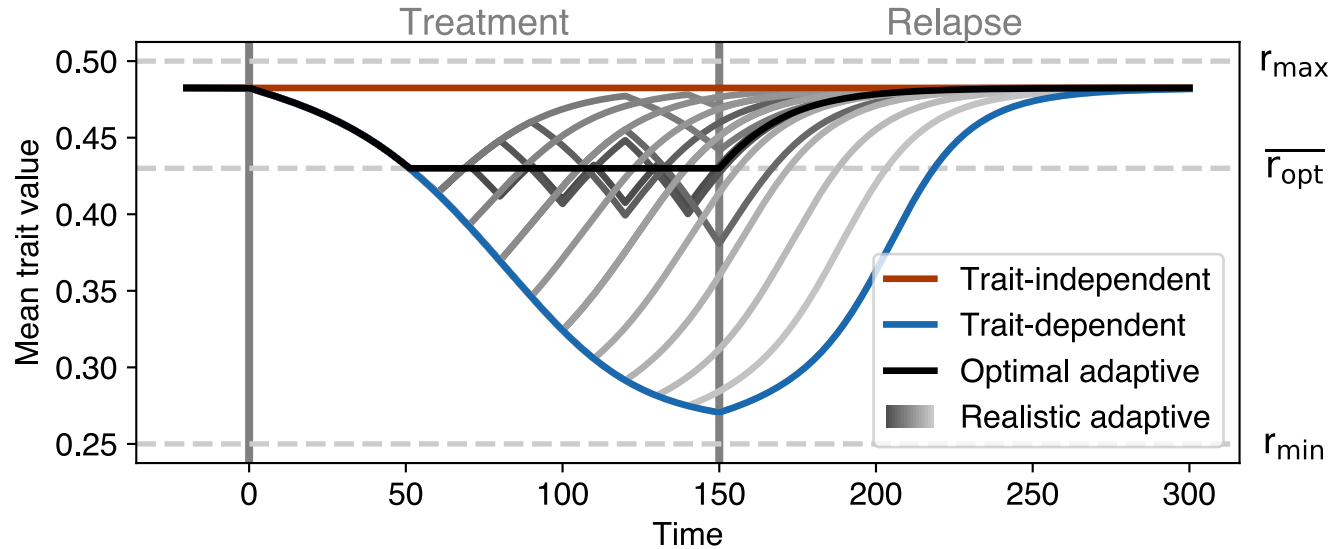

Supplement: S7 Fig — The grey lines correspond to the realistic adaptive scheme with lighter lines showing larger Δt, the difference between them is 10 time units. The optimal adaptive scheme tracks the mean growth rate ropt¯ (Eq 4) where the cancer cell mortality exerted by trait-dependent and trait-independent treatment is equal. The realistic adaptive scheme aims to track ropt¯ and thus oscillates around it. (PDF) [file pcbi.1008702.s009.pdf]
